# Pulmonary Function Testing in Patients With E-Cigarette, or Vaping, Product Use-Associated Lung Injury

**DOI:** 10.7759/cureus.17019

**Published:** 2021-08-09

**Authors:** Xingyu Wei, Annamaria Iakovou, Mina R Makaryus, Sameer Khanijo

**Affiliations:** 1 Internal Medicine, Donald and Barbara Zucker School of Medicine at Hofstra/Northwell/North Shore University Hospital and Long Island Jewish Medical Center, Hempstead, USA; 2 Pulmonary and Critical Care Medicine, Northwell Health, Manhasset, USA; 3 Pulmonary and Critical Care Medicine, Donald and Barbara Zucker School of Medicine at Hofstra/Northwell/North Shore University Hospital and Long Island Jewish Medical Center, Hempstead, USA

**Keywords:** e-cigarette or vaping use-associated lung injury (evali), pulmonary function test, e-cigarette smoking, effects of vaping, lung-injury

## Abstract

In 2019 there was an outbreak of respiratory illnesses amongst people who used E-cigarettes. This phenomenon was labeled 'EVALI' which stands for "Electronic cigarette (E-cigarette), or Vaping, Product Use-Associated Lung Injury" and is a life-threatening illness of the lungs associated with E-cigarette use. It is believed to be caused by certain chemicals in E-cigarette cartridges, such as vitamin E acetate, but the exact pathophysiological mechanism is yet to be elucidated. Since 2019, the CDC has recorded over 2800 cases in the United States with over 60 deaths. Though many people recover from EVALI, the long-term implications on pulmonary health are unknown. The purpose of this retrospective study was to demonstrate the pulmonary function test (PFT) findings in a group of patients who recovered from a diagnosis of EVALI. We reviewed the cases of 23 adult patients who presented to two major academic hospitals of the Northwell Health System with confirmed EVALI and followed up in our outpatient clinics with PFTs. Most patients had significantly reduced diffusion capacity (DLCO) demonstrating loss of functioning alveolar units. Given that average follow-up was over a month after discharge, this leads us to believe that EVALI can lead to persistent lung damage. However, further follow-up would be necessary to identify the full impact of E-cigarette use on the pulmonary function.

## Introduction

In 2019, there was an outbreak of respiratory illnesses amongst people who used E-cigarettes. This phenomenon was labeled 'EVALI' which stands for Electronic cigarette (E-cigarette), or Vaping, Product Use-Associated Lung Injury and is a life-threatening illness of the lungs associated with E-cigarette use. The condition was first identified in the US in early 2019 [1]. Since then, the Centers for Disease Control and Prevention (CDC) has recorded over 2807 cases with 68 deaths confirmed in 29 states and the District of Columbia [2]. 

With numerous flavoring additives available, E-cigarettes are believed to be particularly appealing to a younger demographic [3]. Supporting this popularity, it was estimated that over 2.3 million middle and high schoolers had used a flavored e-cigarette product in 2018 [4]. This represented over 60% of the youth E-cigarette-using population and 8% of all middle and high schoolers [4]. In the initial CDC reports on EVALI, 62% of patients were 18-34 years old and 16% were under 18. The proliferation of EVALI has paralleled this increasing popularity of e-cigarette products among teens and young adults [5]. 

Among the various additives, vitamin E acetate, a diluent used in tetrahydrocannabinol (THC) vaping fluids has been linked to cases of EVALI [6,7]. High levels of vitamin E acetate have been detected in BAL samples taken from patients diagnosed with EVALI, with one study demonstrating vitamin E acetate in BAL for 48 out of 51 patients [6]. Even with these observations, the exact pathophysiology of EVALI has not been established, but it is known that the heating of Vitamin E Acetate generates several toxic compounds, including ketenes, alkenes, and benzenes, which when inhaled can damage the lungs [8]. 

The presentation of EVALI is quite heterogeneous, but commonly manifests with symptoms of respiratory illness, such as pneumonia [1]. Typical respiratory symptoms include cough, shortness of breath, and chest pain. Constitutional and gastrointestinal symptoms are often present as well [9]. While there are no definitive diagnostic criteria the CDC has created definitions of probable and confirmed EVALI. The definition of a confirmed EVALI case includes a respiratory illness requiring hospitalization, use of an e-cigarette, or "dabbing", in the 90 days prior to symptom onset, pulmonary opacities on imaging, the absence of respiratory infections on initial workup, and no plausible alternative diagnoses [5]. However, even with this definition, EVALI remains a diagnosis of exclusion with infectious, autoimmune, and rheumatologic etiologies needing to first be ruled out [10]. 

The CDC has provided guidelines for the evaluation and treatment of EVALI. Patients should undergo testing that includes chest imaging, either radiograph or computed tomography (CT) and an infectious disease workup to exclude alternative diagnoses. In cases of EVALI, imaging often demonstrates peripheral ground-glass opacities [11]. Based on the CDC guidelines, invasive pulmonary sampling, such as bronchoscopy or lung biopsy, are not required and once a diagnosis of EVALI is made, treatment centers around corticosteroid therapy and cessation of e-cigarette product use [12]. 

Given the relatively novel nature of this illness, the long-term impacts of EVALI are yet to be elucidated. In this case series, we review the presentations of various patients diagnosed with EVALI and their subsequent follow-up pulmonary function testing (PFT). We hope to expound on the existing literature of EVALI and provide a better understanding of the effects on pulmonary function in patients diagnosed with EVALI in the northeast region of the US. 

## Materials and methods

This study was approved by the Northwell Health Institutional Review Board (IRB# 19-1001) as a minimal-risk research using data collected for routine clinical practice and the requirement for informed consent was waived. The medical records of adult patients with EVALI who were hospitalized at two tertiary care centers within the Northwell Health System between January 2019 and July 2020 were reviewed. Data were collected retrospectively from the electronic medical record (EMR) and Enterprise Sunrise Clinical Manager-AllScripts for patients with confirmed EVALI who underwent PFTs. 

The cases of EVALI were reviewed to ensure they fit the CDC definitions of confirmed EVALI. This definition included a respiratory illness requiring hospitalization, a history of e-cigarette use or dabbing, abnormal pulmonary imaging and the absence of alternative diagnoses including infection [5].

Patients who met these criteria and had outpatient pulmonary follow-up with pulmonary function testing within the period of data review were included in the analysis. 

Data are reported as medians and ranges. Continuous and categorical variables were summarized using descriptive statistics. The authors investigated the patient’s medical records within the EMR and extracted data on demographics (age, gender), use of vaping additives (either nicotine or THC), and bronchoscopy findings if performed. Follow-up PFT results were extracted from the medical record and all were reviewed for adequacy and met the American Thoracic Society standards for acceptability and reproducibility. Time to testing was subsequently also noted. 

## Results

There were 23 patients who met the inclusion criteria of EVALI diagnosis with subsequent outpatient follow-up and pulmonary function testing. The patients ranged in age from 18 to 52 years old with an average age of 27. The majority of patients were male, 18/23 (78.3%). All patients endorsed using THC-based products while 21.7% also admitted to using nicotine-containing products (Table 1). No other illicit substance use was reported by the patients. There was an average of 42.6 days between date of admission and date of PFT with a wide range (7 to 217 days). The average Body Mass Index (BMI) of patients was 27.7 at the time of admission and 27.8 at the time of PFT. Eight of the 23 patients underwent bronchoscopy while admitted to the hospital. Ten of the 23 patients required high flow nasal cannula or non-invasive ventilatory support. None of the patients required mechanical ventilation.

**Table 1:** Patient demographics. BMI: body mass index; PFT: pulmonary function testing.

Patient characteristics	
Sample size (n)	23
Average age (years)	27 (range: 18-52)
Male	18/23 (78.3%)
Female	5/23 (21.7%)
Vaping of cannabis-related products	23/23 (100%)
Vaping of nicotine-containing products	5/23 (21.7%)
Average BMI prior to admission	27.7
Average BMI on PFT	27.8
Average difference in BMI	0.17
Proportion undergoing bronchoscopy	8/23 (21.7%)
Average days elapsed between admission and PFT	42.6 (range: 7-217)

The PFT findings are described in Table 2. The most common PFT findings were a normal spirometry and lung volumes with a slight reduction in diffusion capacity. The average forced expiratory volume in 1 second (FEV1) was 92.4% (range: 79 to 114). No patients had an FEV1 % < 70% predicted. The average FEV1/FVC was normal at 83.6% (range: 70 to 104). No patients had an FEV1/FVC < 70%. 

**Table 2:** Pulmonary function testing data. FEV1: forced expiratory volume in 1 second; FVC: forced vital capacity; TLC: total lung capacity; RV: residual volume; DLCO: diffusion capacity for carbon monoxide; DLCO/VA Adj: diffusion capacity for carbon monoxide adjusted for alveolar volume; post BD: post bronchodilator.

Parameters	N = 23
Predicted FEV1 (%)	92.4 (79-114)
Predicted FVC (%)	95.0 (70-115)
FEV1/FVC (%)	83.6 (70-104)
TLC (%)	93.1 (71-112)
RV (%)	86.6 (18-149)
DLCO (%)	69.8 (53-91.8)
DLCO/VA Adj	87.2 (67-109)
Bronchodilator testing	N = 11
Post BD FEV1 (%)	95.8 (73-114)
Post BD FVC (%)	98.4 (76-113)
Post BD FEV1/FVC (%)	88.1 (69-115)

Lung volumes were also normal. Average total lung capacity (TLC) was 93.1% and the average residual volume was 86.6%. 

The average diffusion capacity of carbon monoxide was 69.8% (range: 53 to 91.8). Fifteen out of 23 patients had DLCO <75%. Based on the American Thoracic Society criteria for degree of severity [13], the majority of patients, 15/23, had a mild reduction in DLCO. Four patients had a normal DLCO and 4 patients had a moderate reduction in DLCO.

Eleven of the 23 patients had bronchodilator testing at the time of their PFT. Two of 11 patients had a positive response to bronchodilator testing. None of the patients saw a pulmonologist or had PFTs done prior to their hospitalization. None of the 23 patients died during their hospitalization. 

## Discussion

To our knowledge, this study is the largest reported case series of pulmonary function testing in patients diagnosed with EVALI. Our study expands upon two prior cases series which have described a similar finding of reduction in diffusion capacity amongst EVALI patients [9,14]. 

The purpose of this retrospective study was to demonstrate the PFT findings in patients diagnosed with EVALI. Traditional tobacco smoking is characterized by many deleterious effects include, but not limited to, the development of obstructive lung disease and the increased risk of lung cancers. It is now becoming increasingly clear that E-cigarettes may have their own significant effects on long-term pulmonary health. A recently published study has shown that E-cigarette use results in acute alteration in pulmonary function and airway inflammation in stable moderate asthmatic patients [15]. 

In this report, we retrospectively explored a series of EVALI cases. These cases were treated in the Northwell Health System and followed up in associated pulmonary clinics. Treatment approach was conservative with the use of corticosteroids, cessation of the e-cigarette, and use of supplemental oxygen as needed. Similar exploration of this topic has been undertaken by various health systems across America, but we believe our work extends on this body of literature by exploring the presentation of cases in the NYC (New York City) and Long Island region. The demographic characteristics of the population studied also resemble those of several previous cases series and reports in terms of a younger age group and male predominance [1,5]. 

Our findings demonstrate that while lung function was preserved in the post-EVALI state, there was a notable mild reduction in the diffusion capacity which persisted after hospital discharge suggesting that there was lung damage and loss of functioning alveolar units. The majority of patients demonstrated a mild reduction in DLCO. Interestingly, there were 2 patients who had PFTs performed more than 100 days after their hospitalization (112 days and 217 days, respectively) and both patients had a persistent mild reduction in DLCO. Both obstructive and restrictive lung diseases can cause a diminished diffusion capacity and the exact etiology from these EVALI cases is not yet known and will require long-term follow-up. 

Limitations in our study include all the ones inherent to a retrospective analysis such as the inability to attribute causation and the lack of a control group. There was a selection bias in this cohort as it includes only patients who had pulmonary follow-up with PFTs. There was no standard time to follow-up for PFTs. While the majority of PFTs were performed within the first two months after hospitalization there was a wide range of dates of follow-up which may affect the results. Our ability to obtain additional longitudinal data was disrupted due to the COVID pandemic. A longer series of PFTs over time may reveal additional abnormalities. In addition, our patient population was younger without a history of any significant underlying lung disease which may explain the relatively normal pulmonary functions noted. 

## Conclusions

We describe a case series of patients with EVALI who had subsequent pulmonary function testing. We believe that the initial reduction in diffusion capacity is consistent with the parenchymal lung damage that has been noted in EVALI patients. The longitudinal impact of e-cigarette use, or vaping, on pulmonary function is still unknown. We believe further follow-up and study are necessary to uncover potential long-term sequelae. If such long-term sequelae become apparent, it may then be necessary to consider the utility of screening measures, such as further follow-up PFTs.
